# Transcriptomic Analysis Provides Novel Insights into the Heat Stress-Induced Response in *Codonopsis tangshen*

**DOI:** 10.3390/life13010168

**Published:** 2023-01-06

**Authors:** Xiaogang Jiang, Misganaw Wassie, Wuxian Zhou, Hua Wang, Jinwen You, Guangjing Ma, Meide Zhang

**Affiliations:** 1 Key Laboratory of Biology and Cultivation of Herb Medicine, Ministry of Agriculture and Rural Affairs, Institute of Chinese Herbal Medicines, Hubei Academy of Agricultural Sciences, Enshi 445000, China; 2 CAS Key Laboratory of Plant Germplasm Enhancement and Specialty Agriculture, Wuhan Botanical Garden, The Innovative Academy of Seed Design, Chinese Academy of Sciences, Wuhan 430074, China

**Keywords:** *Codonopsis tangshen*, heat-stress, heat-responsive genes, RNA-sequencing, transcriptome

## Abstract

*Codonopsis tangshen* Oliv (*C. tangshen*) is a valuable traditional Chinese medicinal herb with tremendous health benefits. However, the growth and development of *C. tangshen* are seriously affected by high temperatures. Therefore, understanding the molecular responses of *C. tangshen* to high-temperature stress is imperative to improve its thermotolerance. Here, RNA-Seq analysis was performed to investigate the genome-wide transcriptional changes in *C. tangshen* in response to short-term heat stress. Heat stress significantly damages membrane stability and chlorophyll biosynthesis in *C. tangshen*, as evidenced by pronounced malonaldehyde (MDA), electrolyte leakage (EL), and reduced chlorophyll content. Transcriptome analysis showed that 2691 differentially expressed genes (DEGs) were identified, including 1809 upregulated and 882 downregulated. Functional annotations revealed that the DEGs were mainly related to heat shock proteins (HSPs), ROS-scavenging enzymes, calcium-dependent protein kinases (CDPK), HSP-HSP network, hormone signaling transduction pathway, and transcription factors such as bHLHs, bZIPs, MYBs, WRKYs, and NACs. These heat-responsive candidate genes and TFs could significantly regulate heat stress tolerance in *C. tangshen*. Overall, this study could provide new insights for understanding the underlying molecular mechanisms of thermotolerance in *C. tangshen*.

## 1. Introduction

*Codonopsis tangshen* Oliv is a perennial herbaceous plant belonging to the family Campanulaceae. *C. tangshen* is widely used in traditional Chinese medicine due to its tremendous health benefits. Traditional medicine prepared from the dried roots of *C. tangshen* is commonly used to strengthen the spleen and lungs, nourish the blood, and maintain body fluids [1]. The main medicinal constituents of *C. tangshen* include polysaccharides, saponins, alkaloids, sesquiterpenes, glycosides, polyphenolic polyacetylenes, etc. [2,3].

Modern pharmacology and clinical studies also demonstrated that the main bioactive components of *C. tangshen* have antioxidant, antibiosis, anti-inflammatory, antitumor, and immune enhancement effects [4,5,6,7,8,9]. *C. tangshen* is mainly cultivated in Chongqing, Hubei, Sichuan, Hunan, Guizhou, and Shanxi provinces [10]. *C. tangshen* grows best in high-altitude areas with mild and cool climates [11]. However, a temperature above 30 °C could inhibit the growth and development of *C. tangshen*, thus reducing yield and quality [12], indicating that extremely high temperature is a severe problem for the cultivation of *C. tangshen*.

It is well documented that short-term or long-term heat stress exposure could significantly decline growth, development, and yield in many plant species. For instance, a 1 °C rise could reduce crop yields by 17% during the growing season [13]. Continuous heat stress could delay panicle emergence and reduce plant height, seed setting rate, seed size, and harvest index in sorghum [14]. In wheat, heat stress reduces grain yield by affecting various physiological, molecular, and biological processes [15].

Additionally, Ma et al. (2020) [16] demonstrated that exposure of *Pinellia ternata* to heat stress reduced cell membrane stability, antioxidant capacity, and chlorophyll content, thereby declining plant productivity. Heat stress inhibited the photosynthetic apparatus of *Solanum lycopersicum* L. and decreased photosynthetic efficiency [17]. A recent study also indicated that high temperature decreased trace elements, protein, soluble sugar, and anti-oxidation ability in cauliflower (*Brassica oleracea var. botrytis* L.) [18].

The effect of heat stress is mainly related to the over-accumulation of heat stress-induced reactive oxygen species (ROS), which further leads to oxidative stress. Accumulating evidence reveals that oxidative stress could damage membrane stability and integrity, thus, leading to electrolyte leakage and membrane lipid peroxidation. Meanwhile, plants also activate enzymatic antioxidant defense machinery, including glutathione S-transferase (GST), pheophorbide a oxygenase (PAO), superoxide dismutase (SOD), catalase (CAT), and peroxidase (POD) to scavenge the over-accumulated ROSs [19,20]. Plant hormone signaling transduction could also play a remarkable role in heat stress response. Several ABA metabolism and auxin-associated genes have been identified in *A. thaliana* and rice exposed to heat stress, respectively [21,22].

In recent decades, outstanding progress has been made in understanding plants’ response to heat stress. To this end, the regulatory mechanisms involved in acquiring thermotolerance and long-term adaptation to heat stress have been elucidated [23,24]. At the molecular level, the heat stress response is mainly associated with the activation of HSF (heat shock transcription factor) and HSP (heat shock protein) networks that further regulate heat shock proteins (HSPs), including HSP20s, HSP70s, HSP90s, and HSP100s [25]. These heat-induced proteins provide various functions, including protecting proteins from heat stress-induced degradation and assisting in maintaining their native structures [26]. Additionally, hormone-metabolism mechanisms are crucial in response to heat stress [27,28].

Despite several studies on understanding the mechanisms of heat tolerance in a wide range of plant species, how heat stress affects and reprograms the physiological processes of *C. tangshen*, especially at the transcriptome level, remains largely unknown. RNA-seq is a high-throughput sequencing technology that can also be used to study plants without a reference genome [29,30]. In this study, RNA-seq was performed to investigate how *C. tangshen* responded to short-term heat stress at the transcriptome level. In uncovering the mechanisms of heat stress response in *C. tangshen*, key DEGs and metabolic pathways significantly regulated by heat stress were identified. This study lays a foundation for elucidating the underlying molecular mechanisms of heat stress response and provides a basis for improving the thermotolerance of *C. tangshen*.

## 2. Materials and Methods

### 2.1. Plant Materials and Sample Collection

The fresh roots of *C. tangshen* were planted in plastic pots (50 cm length, 21 cm width, and 15 cm depth) filled with appropriate nutrient soil. The pots were kept in an incubator with light/dark of 14/10 h, a temperature of 22/18 °C, photosynthetic photon flux density of 360 mmol m^−2^ s^−1^, and watered every two days with half-strength Hoagland solution. After 40 days of growth, the pots were divided into two groups (3 pots for each group) and placed into different growth incubators with the same condition except for temperature. The control group was kept at 22/18 °C (day/night) (CK), while the heat stress group (HT) was exposed to high-temperature treatment at 42 °C. After 0.5 h of treatment, young leaf samples were collected with three replicates for each group and are used for transcriptome sequencing. Short-term (0.5 h) heat stress had no significant effect on the morphological and physiological traits of *C. tangshen*. Thus, heat stress was extended to 7 d, where the plants were exposed to high-temperature treatment at 38/35 ℃ (day/night). Plants were photographed for phenotypic analysis and leaf samples were collected from control and heat-treated plants for physiological and biochemical analysis. All the treatments were repeated in three biological replications.

### 2.2. RNA Extraction and cDNA Library Preparation

Total RNA was extracted from the leaf samples using TRIzol reagent (Invitrogen, Carlsbad, CA, USA). The quality and quantity of RNA samples were monitored using the Nano Photometer spectrophotometer (IMPLEN, Westlake Village, CA, USA) to ensure high-quality samples for transcriptome sequencing. About 1 μg RNA per sample was used to construct the paired-end libraries using NEBNext^®^ Ultra™ RNA Library Prep Kit for Illumina^®^ (NEB, Ipswich, MA, USA) according to the manufacturer’s instructions. First-strand cDNA was generated using random hexamer primer and M-MuLV Reverse Transcriptase, followed by synthesizing second-strand cDNA using DNA Polymerase I and RNase H. Then, fragmentation was performed using divalent cations under increased temperature. After adenylation of 3’ ends of DNA fragments, adaptor sequences with hairpin loop structure were ligated to the fragments. Subsequently, the fragments about 240 bp in length were purified using the AMPure XP system (Beckman Coulter, Beverly, USA). Then, PCR was carried out using Phusion High-Fidelity DNA polymerase, Universal PCR primers, and Index Primer. Subsequently, PCR products were purified, and the quality of the libraries was checked with the Agilent Bioanalyzer 2100 system. Six libraries were prepared using the NEBNext^®^Ultra™ RNA Library Prep Kit for Illumina^®^ (NEB, USA), following the manufacturer’s recommendations. Sequencing was performed using an Illumina Hiseq 2000 platform in Novogene Co., Ltd. (Beijing, China).

### 2.3. Measurement of Physiological Properties

Physiological properties, including chlorophyll content, electrolyte leakage (EL), and malonaldehyde (MDA) content were measured and analyzed according to a previous study [16]. For chlorophyll content analysis, 0.1 g leaves were collected and immersed in 95% ethanol. The samples were kept in the dark for 48 h and absorbance of the chlorophyll extract was read at 665 and 649 nm using a spectrophotometer (UV2600, UNIC, Shanghai, China). The chlorophyll content was calculated according to the formula described in a previous study [16].

For EL analysis, 0.5 g fresh leaves were collected and washed with distilled water to remove debris. Then, 15 mL distilled water was added to each tube and kept in a shaker incubator at 200 rpm for 6 h at 28 °C. Subsequently, the initial conductivity (EL1) was measured using a conductivity meter (JENCO-3173, Jenco Instruments, Inc., San Diego, CA, USA). Then, samples were autoclaved at 121 °C for 30 min. Immediately, the tubes were cooled to room temperature and the second conductivity (EL2) was measured. The relative EL was calculated by using the formula:

Relative EL (%) = (EL1/EL2) × 100

For MDA content determination, 0.3 g frozen leaves were used to prepare the crude enzyme extract. Leaves were ground into powder in liquid nitrogen using a pre-chilled mortar and pestle (4 °C), homogenized with sodium phosphate buffer (PBS), pH 7.4, and centrifuged at 12,000 rpm for 20 min at 4 °C. The supernatant was used to measure the MDA content. A total of 1 mL extract was mixed with a 2 mL reaction solution containing 20% (*v*/*v*) trichloroacetic acid and 0.5% (*v*/*v*) thiobarbituric acid. The mixture was boiled in a water bath for 30 min, cooled to room temperature, and centrifuged at 12,000× *g* for 10 min at 20 °C. The absorbance was read at 532 and 600 nm with a spectrophotometer (UV2600, UNIC, Shanghai, China). The MDA content was determined using the following formula: MDA (nmol g^−1^ FW) = [(A532 − A600) × V × 1000/ε] × W

Where A600 and A532 are the absorbances at 600 and 532 nm, respectively, ε is the specific extinction coefficient (155 mM cm^−1^), V is the volume of crushing medium and W is the fresh weight of leaves (g).

The physiological traits analysis was performed with three biological replicates, and one-way ANOVA was used to analyze differences between the control and heat-treated group.

### 2.4. Bioinformatics Analysis

#### 2.4.1. Processing and Assembling of Illumina Reads

Paired-end reads were generated from the six libraries. Clean reads were obtained by removing low-quality reads and reads containing adapters or poly-N. Subsequently, the Q20, Q30, GC-content, and sequence duplication levels were analyzed. The downstream analyses were carried out using high-quality clean data. Transcriptome assembly was performed using the Trinity version 2.8.5 with all parameters set to default [31].

#### 2.4.2. Gene Functional Annotation

Gene function was annotated by using the following databases: NR (NCBI non-redundant protein sequences) (http://www.ncbi.nlm.nih.gov, accessed on 11 March 2021), Pfam (Protein family) (http://pfam.sanger.ac.uk/, accessed on 11 March 2021), GO (Gene Ontology) (http://geneontology.org/, accessed on 11 March 2021), KOG/COG (Clusters of Orthologous Groups of Proteins) (ftp://ftp.ncbi.nih.gov/pub/COG/, accessed on 11 March 2021), Swiss-Prot (a manually annotated and reviewed protein sequence database) (http://www.ebi.ac.uk/uniprot/, accessed on 11 March 2021), and KEGG (Kyoto Encyclopedia of Genes and Genomes) (http://www.genome.jp/kegg/, accessed on 11 March 2021).

#### 2.4.3. Differential Gene Expression Analysis

For each sample, gene expression levels were evaluated by RSEM [32]. Briefly, clean data were mapped back onto the assembled transcriptome, and then a read count for each gene was obtained based on the mapping results. Then, differential expression analysis of two groups was performed with DESeq2 version 3.11 [33] using a model based on the negative binomial distribution. The *p* values were adjusted to control the false discovery rate with the Benjamini and Hochberg methods. P-adj < 0.05 and |log2foldchange| > 1 were set as the cutoff value to determine significantly differentially expressed genes (DEGs).

#### 2.4.4. GO and KEGG Pathway Enrichment Analysis

The clusterProfiler 4.0 (https://github.com/GuangchuangYu/clusterProfiler, accessed on 11 March 2021) was used for GO and KEGG pathways enrichment analysis of the differentially expressed genes (DEGs) [34]. Adjusted *p* value < 0.05 (as predicted by clusterProfiler software) was used as the threshold for the GO and KEGG pathway enrichment analysis.

#### 2.4.5. Clustering of the DEGs

Hierarchical clustering was performed to observe the expression patterns of DEGs. The RPKM counts for each unigene were clustered using Cluster 3.0 (http://bonsai.hgc.jp/~mdehoon/software/cluster/, accessed on 11 March 2021) [35], and JAVA Treeview 3.0 was used to visualize the results (https://jtreeview.sourceforge.net/, accessed on 11 March 2021) [36].

### 2.5. Validation of RNA-Seq Data by Real-Time Quantitative PCR

To validate the RNA-Seq results, real-time quantitative RT-PCR was performed for selected target genes using the previous method [37]. Briefly, total RNA was extracted from frozen leaves using the Trizol reagent (Invitrogen, Carlsbad, CA, USA) according to the manufacturer’s instructions. The quality of RNA extract was monitored on 1% agarose gels, and also checked using the NanoPhotometer^®^ spectrophotometer (IMPLEN, Westlake Village, CA, USA). A total of 2 µg of RNA extract was used to prepare cDNA for each sample using RevertAid First Strand cDNA Synthesis Kit (Invitrogen, Carlsbad, CA, USA). The PCR reaction was performed using a total of 20 μL consisting of 10 μL of SYBR mix, 0.5 μL of each primer, 2 μL of cDNA, and 7μL dd water. The ABI StepOne Plus Real-Time PCR system (Applied Biosystems, Foster City, CA, USA) was used for PCR reaction. Melting curve analysis was used to check the specificity of the amplified PCR product after each PCR reaction. The *glyceraldehyde-3-phosphate dehydrogenase* (*GAPDH*) gene was used as a housekeeping control. The primers used in this study are listed in Table S1. The 2^−ΔΔCT^ method was used to determine the relative expression of genes [38]. One-way ANOVA was applied to analyze the statistical differences between different groups.

## 3. Results

### 3.1. Effect of Heat Stress on Physiological and Biochemical Traits

**Figure 1:**
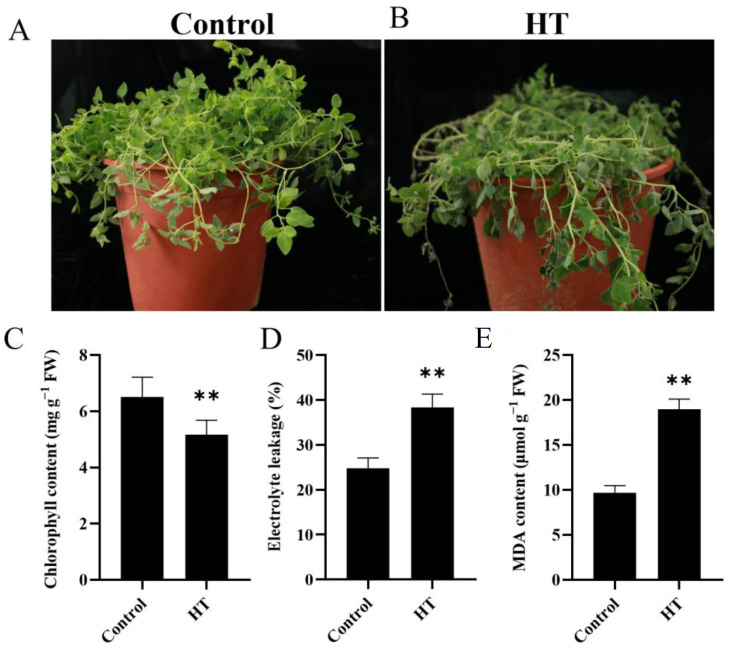
Phenotypic, physiological and biochemical analysis of *C. tangshen* in response to heat stress. (**A**): Phenotypes of *C. tangshen* seedlings under normal conditions; (**B**): phenotypes of *C. tangshen* seedlings after 7 d heat treatment; (**C**): chlorophyll content; (**D**): electrolyte leakage (EL); (**E**): malondialdehyde (MDA) content. Four weeks old plants were kept under optimal moderate (22/18 °C) or high (38/35 °C) temperature treatments. There were three repeats for this experiment. The asterisks indicate significant differences based on one-way ANOVA with Fisher’s LSD test (**, *p* < 0.01).

Short-term (0.5 h) heat stress treatment did not significantly affect the morphology of *C. tangshen*. However, long-term heat stress exposure induced morphological and physiological alterations in *C. tangshen.* After 7 d of heat stress, the leaves of *C. tangshen* showed wilting and became withered (Figure 1A,B). Heat stress also significantly decreased chlorophyll content by 20.71% relative to the control (Figure 1C). Heat stress dramatically increased EL and MDA content by 54.67% and 95.74%, respectively, relative to the control (Figure 1D,E).

### 3.2. Illumina Sequencing and De Novo Assembly

Six cDNA libraries were constructed and sequenced from the leaves of *C. tangshen*. After quality control, a total of 44,915,008, 47,205,020, 52,814,730, 50,974,272, 44,953,364, and 49,951,766 clean reads were obtained from control (CK) and heat treatment (HT) libraries, respectively. The average clean reads of CK and Ht were 48,311,586 and 48,626,467, respectively. In addition, the average GC contents were 44.79% for CKs and 45.30% for Ht. The mean Q20 values were 97.99% and 98.11%, and the Q30 values were 93.79% and 94.12% for CK and Ht, respectively, indicating the high quality of the transcriptome sequencing results (Table 1). The raw sequencing data have been submitted to the Genome Sequence Archive (GSA) database at the BIG Sub (CRA004261). In addition, de novo assembly was carried out and identified 74,144 transcripts and 32,719 unigenes with an average length of 2346 bp and 2472 bp, respectively. All the unigenes are listed in Table S2.

### 3.3. Functional Annotations of C. tangshen Unigenes

**Figure 2:**
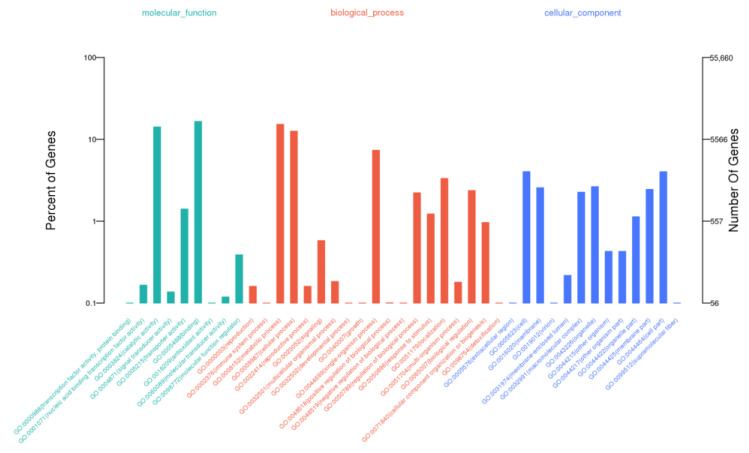
GO classification of assembled unigenes. The unigenes were annotated into the three main GO categories: biological process, cellular component, and molecular function.

The functional annotations of unigenes were performed by searching against several databases, including KOG (Clusters of Orthologous Groups of Proteins), GO (Gene Ontology), KO (KEGG Ortholog Database), NR (NCBI non-redundant protein sequences), Swiss-Prot (a manually annotated and reviewed protein sequence database) (Table 2). A total of 19,546 (59.7%) unigenes had hits in the GO database and were classified into three GO categories, including “biological process (BP),” “cellular component (CC),” and “molecular function (MF) (Figure 2).

**Figure 3:**
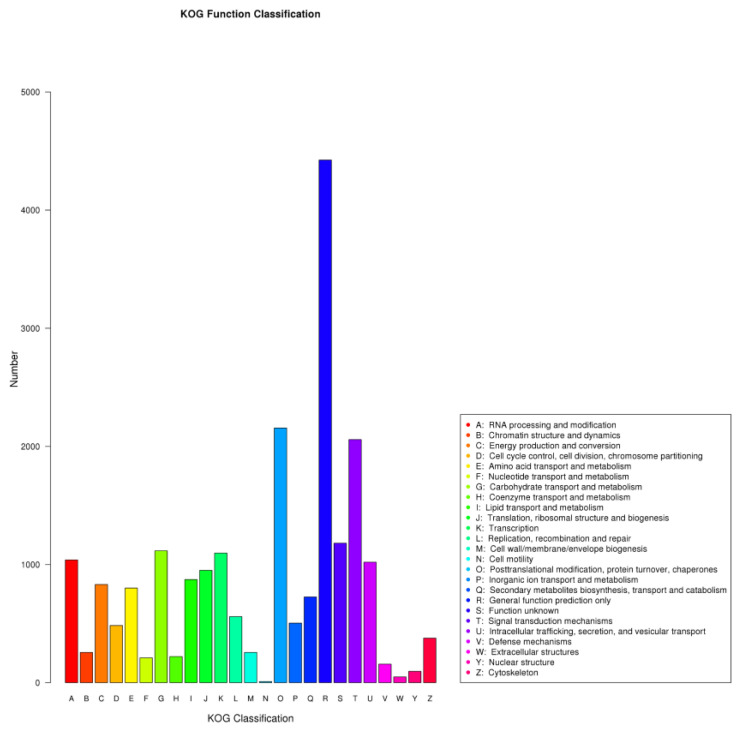
KOG annotation of assembled unigenes. The unigenes were annotated into twenty-five main classifications, including RNA processing and modification, chromatin structure and dynamics, energy production and conversion, amino acid transport, and metabolism, etc.

In the “BP” category, the highly enriched terms were “metabolic process,” “cellular process,” and “single-organism process.” The “CC” category mainly comprised unigenes related to “cell,” “cell part,” and “organelle.” Additionally, “the catalytic activity,” “transporter activity,” and “binding” were significantly enriched in the “MF” category (Figure 2, Table S3). Moreover, 19,217 (58.7%) unigenes were annotated using the KOG database and classified into 25 different functional classes (Figure 3). The main categories include “General function prediction only,” “Posttranslational modification, protein turnover, chaperones,” and “Signal transduction mechanisms,” each comprising 4424, 2156, and 2058 unigenes, respectively.

**Figure 4:**
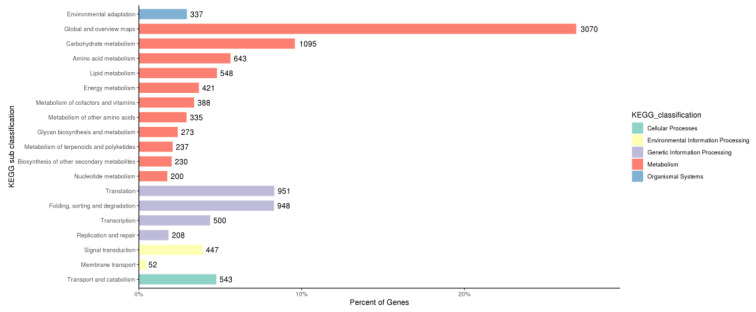
KEGG classification of unigenes. The unigenes were annotated into five main classifications: cellular processes, environmental information processing, genetic information processing, metabolism, and organismal systems.

In addition, all the unigenes were searched against the KEGG database (Figure 3). A total of 12,562 unigenes were assigned to five KEGG pathways, including “cellular processes” (A), “environmental information processing” (B), “genetic information processing” (C), “metabolism” (D), and “organismal systems” (E) (Figure 4). Category A comprised only one subcategory, “Transport and catabolism” (543 unigenes). Category B consisted of two subcategories: “Signal transduction” (447 unigenes) and “Membrane transport” (52 unigenes). Two subcategories dominated Category C: “Translation” (951 unigenes) and “Folding, sorting, and degradation” (948 unigenes). Category D was mainly represented by two subcategories: “Global and overview maps” (3070 unigenes) and “Carbohydrate metabolism” (1095 unigenes). The “environmental adaptation” was the only enriched subcategory in category E.

### 3.4. Analysis of DEGs in Response to Heat Stress in C. tangshen

To obtain insights into the transcriptomic response of *C. tangshen* to heat stress, we further analyzed the differentially expressed genes (DEGs) list that was identified using the DESeq package using the CK (control samples) and HT (heat-treated samples). A total of 2691 DEGs were identified, including 1809 upregulated and 882 downregulated. The expression and annotation of DEGs are shown in Table S4 and Table S5, respectively.

**Figure 5:**
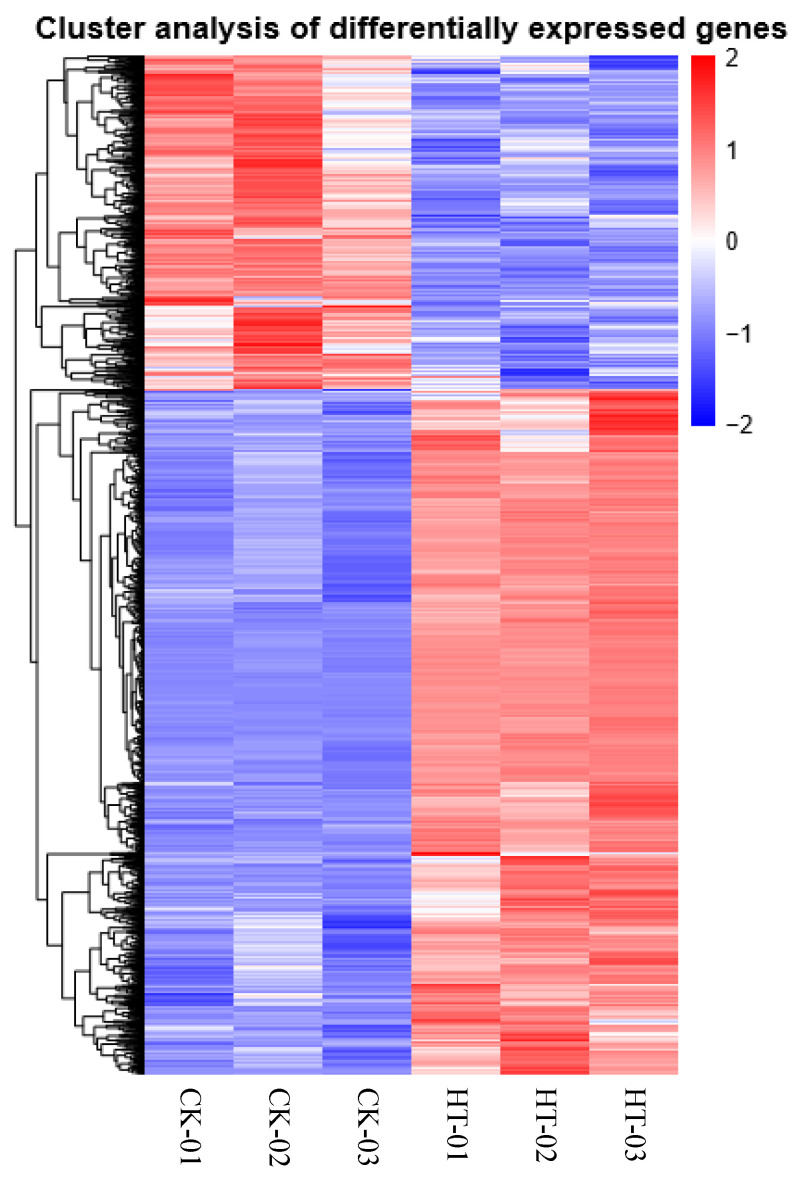
Hierarchical clustering analysis of differentially expressed genes. The red color represents high expression levels, and blue represents low expression levels. The values besides the colors represented log_2_ (FPKM + 1).

Moreover, the DEGs from the three control and heat treatment replicates were clustered using the hierarchical clustering method (Figure 5). The DEGs were classified into two groups and showed contrasting expression profiles between CK and heat treatment. Genes with similar expression levels were preferentially clustered together. There were no significant differences in the expression of DEGs between replicates.

### 3.5. GO and KEGG Enrichment Analyses of DEGs

**Figure 6:**
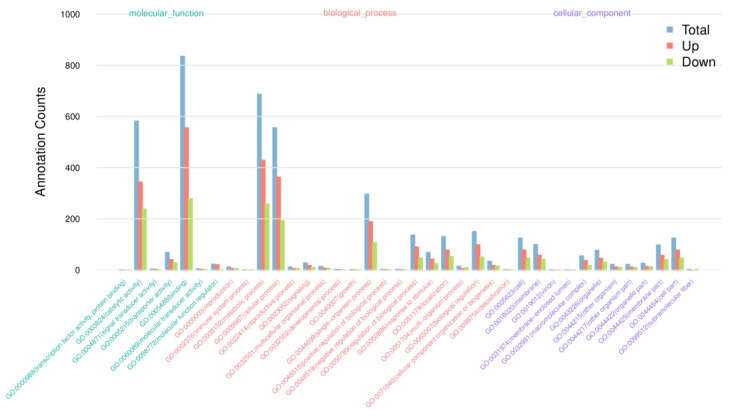
The statistics results of enriched GO terms. The unigenes were annotated into the three GO categories: biological process, cellular component, and molecular function.

GO analysis was performed to investigate the functions of DEGs. As shown in Figure 6, Table S6, DEGs were classified into three GO categories, MF, BP, and CC. The highly enriched MF GO terms were unfolded protein binding, chaperone binding, DNA binding transcription factor activity, ATPase activator activity, channel activity, and heat shock protein binding. In the BP category, protein folding, protein refolding, regulation of pH, sodium ion transport, lipid catabolic process, photosynthetic electron transport chain, and “photosynthesis, light reaction” were significantly enriched. However, we did not find significantly enriched GO terms in the CC category.

Furthermore, to investigate the metabolic pathways regulated in response to heat stress in *C. tangshen*, DEGs were systematically subjected to the KEGG pathway enrichment analysis (Figure S2, Table S7). We found 30 enriched metabolic pathways. Interestingly, the upregulated DEGs were mainly enriched into plant–pathogen interaction (67 DEGs) and protein processing in the endoplasmic reticulum (123 DEGs) (Figure S3, Table S8). While downregulated DEGs were significantly enriched into starch and sucrose metabolism (15 DEGs), phenylpropanoid biosynthesis (11 DEGs), fatty acid elongation (5 DEGs), and plant–pathogen interaction (16 DEGs) (Figure S4, Table S9).

### 3.6. DEGs Related to Short-Term Heat Stress in C. tangshen

To identify heat stress-responsive candidate genes, we focused on genes related to HSF-dependent pathways, ROS scavenging enzymes, plant hormone signaling transduction, and transcription factors, including bHLH, bZIP, MYB, WRKY, and NAC. When exposed to heat stress, the increased expression of HSPs usually form a complex with the HSF [39]. We found 130 HSPs and 8 HSFs enriched by heat stress-induced upregulated genes in the HSF-dependent pathway. ROS levels increased significantly in plants exposed to high temperatures [40]. We identified six GSTs and one PAO significantly upregulated genes involved in ROS scavenging. Furthermore, genes related to plant hormone signaling transduction were significantly induced by heat treatment, including eleven ERFs, two ABFs, and one PYL. Calcium-dependent protein kinases (CDPKs) genes are critical components in the heat stress-mediated calcium signal transduction pathway [41]. Here, a total of 15 CPKs and 5 calmodulin-like proteins (CMLs) were significantly upregulated by heat stress in *C. tangshen* (Table 3).

**Figure 7:**
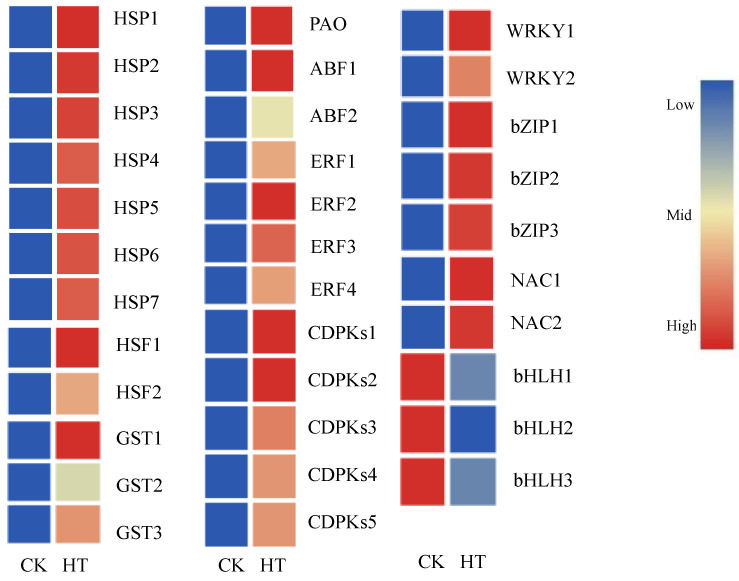
Clustering of the differentially expressed genes related to the representative gene category in heat treatment compared with CK. Red color denoted genes with high expression levels, while blue color indicated genes with low expression levels. The relative expression levels of DEGs were calculated using the log2 ratio.

In addition, heat-responsive transcription factors (TFs), including bHLH, bZIP, WRKY, and NAC, were identified in *C. tangshen*. Out of the 10 bZIP genes, 8 were significantly upregulated by heat treatment compared to the control (Table 4). Additionally, 14 DEGs encoding the NAC TFs were identified, of which 11 were significantly upregulated, while the other 3 were remarkably downregulated after heat treatment (Table 5). We also identified nine WRKY genes that were significantly upregulated by heat stress (Table 6). Moreover, three bHLH genes were identified and remarkably downregulated upon heat stress (Table 7). The clustering of the representative DEGs related to the different gene categories in heat treatment compared with CK is shown in Figure 7.

### 3.7. Validation of the DEGs by qRT-PCR

**Figure 8:**
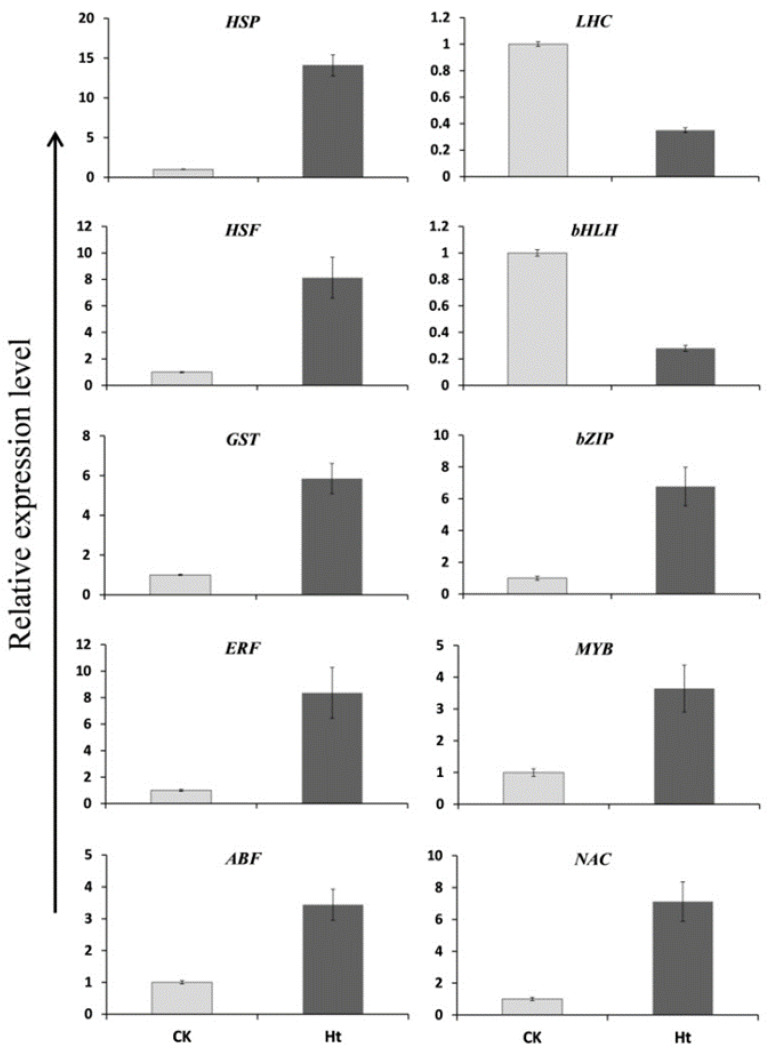
The qRT-PCR analysis of 10 DEGs in the leaves of heat-treated (Ht) and control (CK) plants.

The RNA-Seq results were validated by analyzing the expression of ten unigenes related to TFs or stress responses using qRT-PCR. The transcriptional level of three stress-responsive unigenes (*HSP*, *HSF*, and *GST*) was significantly increased in the Ht group compared to the CK group (Figure 8). Six TFs genes were also checked. Five TFs were markedly upregulated by heat stress (*ERF*, *ABF*, *bZIP, MYB*, and *NAC*), and only one (*bHLH*) was downregulated by heat stress. Additionally, the expression of a light-harvesting chlorophyll a/b binding protein (LHC) gene was inhibited by heat stress. In summary, the expression profiles of these unigenes from the qRT-PCR were consistent with that from the RNA-Seq, indicating that the RNA-Seq data were reliable and accurate.

## 4. Discussion

### 4.1. Heat Stress Regulates Physiological and Biochemical Traits in C. tangshen

Heat stress is a severe abiotic factor limiting plants’ growth and development. In this study, we demonstrated the heat stress-induced biochemical and transcriptomic responses of *C. tangshen*. Transcriptomic analysis was carried out after short-term heat stress (0.5 h) treatment. Extended heat stress for 7 d caused a noticeable phenotypic change in *C. tangshen*, including leaf wilting. Electrolyte leakage and lipid peroxidation have been used to study heat stress-induced membrane damage in plants [42,43]. Physiological and biochemical analysis showed that *C. tangshen* was severely affected by 7 d heat stress treatment, evidenced by pronounced levels of electrolyte leakage and lipid peroxidation. Additionally, chlorophyll content was significantly reduced by heat stress. Our results agreed with the findings reported in *P. ternate* [16]. Together, these results indicate that heat stress is a significant limiting factor for the growth and development of *C. tangshen.* Thus, identifying key heat-responsive genes and understanding the underlying molecular mechanisms could be crucial to improve the thermotolerance of *C. tangshen.*

### 4.2. Transcriptomic Response of C. tangshen to Heat Stress

Plants reprogram transcriptomic profiles and develop molecular mechanisms to cope with heat stress [44,45,46]. In this study, we conducted RNA-seq to understand the molecular mechanisms of heat stress response in *C. tangshen* at the transcriptome level. Here, 2691 DEGs were identified, including 1809 upregulated and 882 downregulated. Further analysis demonstrated that most of the DEGs induced by heat stress were related to the HSF–HSP pathway, ROS scavenging enzymes, plant hormone signaling transduction, and TFs, including bZIP, MYB, NAC, WRKY, and bHLH, which have been reported in previous studies [25,27,47,48]. This study is the first report to investigate the transcriptomic profiles of *C. tangshen* in response to heat stress. Moreover, we identified candidate heat-responsive genes and transcription factors that could play a crucial role in improving the thermotolerance of *C. tangshen*. Overall, our results provide essential information for the functional characterization of heat-responsive genes in *C. tangshen*.

### 4.3. ROS–Scavenging Activity and HSF–HSP Network Involved in HSR

Heat stress induces excessive production of reactive oxygen species (ROS) in plant cells, leading to oxidative stress, which further causes DNA damage, protein denaturation, and impairment of cell membranes [49]. ROS-scavenging enzymes, such as GSTs and APXs, play a crucial role in heat stress tolerance in plants [50]. In this study, six *GST* and two *APX* genes were significantly induced in response to heat stress, indicating that these genes could be involved in scavenging ROS and protecting *C. tangshen* from heat stress-induced damages.

The HSF–HSP network is activated to protect plant cells against heat stress-induced damages [51]. In *Arabidopsis*, HSPs, including *Hsp* 100, *Hsp90*, *Hsp70*, and *Hsp60*, were significantly induced to provide mechanisms against heat stress [52]. In this study, heat stress induced the expression of HSF and HSP genes in *C. tangshen.* For instance, 8 *HSFs* and 130 *HSPs* genes, including *HSP20*, *HSP70*, and *HSP90* were, significantly upregulated by heat stress. Previous studies showed that HSP proteins could be crucial in improving heat tolerance in plants. Overexpression of *HSP70* from *Brassica campestris* improved heat tolerance in tobacco [53]. Additionally, overexpression of a peony *HSP70* enhanced heat tolerance in transgenic *Arabidopsis* [54]. In addition, *GmHsp90A2* from soybean was identified as a positive player of heat tolerance [51]. Thus, *HSP70* and *HSP90* could be key genes to improve the thermotolerance of *C. tangshen.* Meanwhile, further study is required to investigate how HSPs and HSFs function in *C. tangshen* under heat stress.

### 4.4. DEGs Related to Plant Hormone Signal Transduction

**Figure 9:**
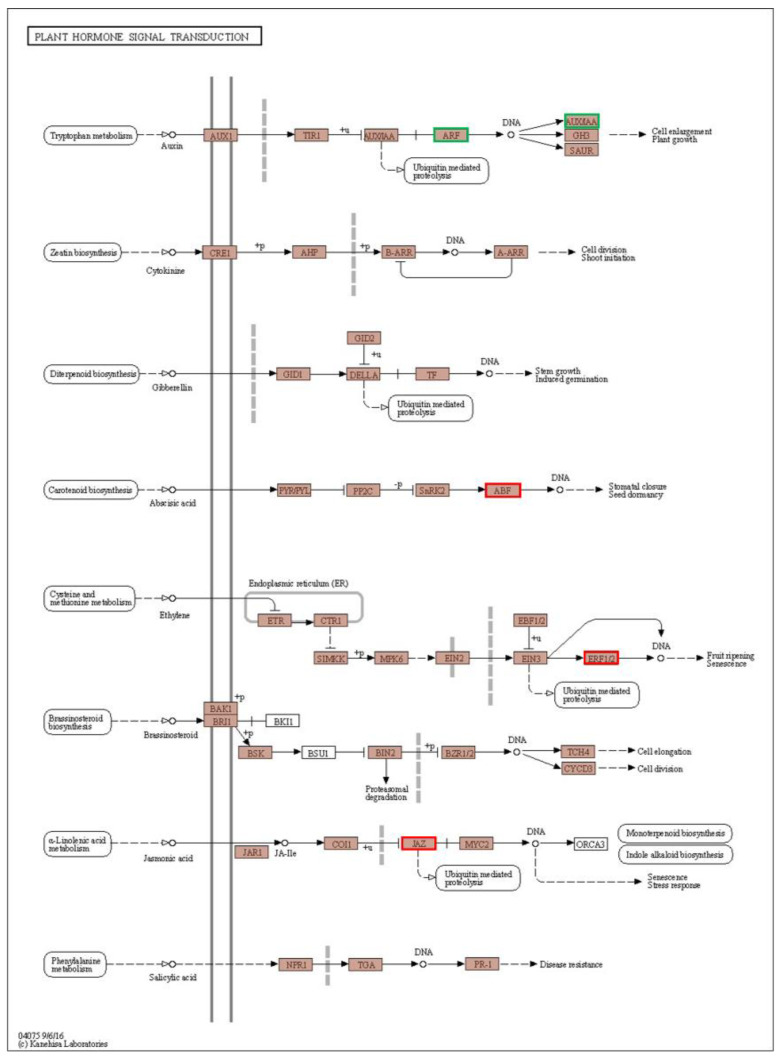
Differentially expressed genes (DEGs) related to plant hormone signal transduction in Ht vs. CK. The green frame indicated the downregulated gene, and red frame denoted the upregulated gene.

The phytohormones, including ABA, ethylene, and brassinosteroids, play crucial roles in plants’ growth development and heat stress response [53,54,55,56,57,58,59]. Ethylene-responsive transcription factors (ERFs) are a transcriptional subfamily downstream of the ethylene signaling pathway [60,61]. ERFs are involved in floral organ development, fruit ripening, abiotic stresses, hormonal signal transduction, and pathogenesis [61,62]. Most *ERFs* were significantly upregulated in plants treated with heat stress [63]. In this study, most *ERF* genes were induced in response to heat stress (Figure 9), suggesting that ERFs might function in protecting *C. tangshen* from heat stress damage. ABFs constitute a significant transcription factor in the ABA signaling pathway that activates ABA/stress-responsive genes in response to abiotic stress [64]. The binding of ABA to PYL proteins inhibits protein phosphatase type 2Cs (PP2Cs) activity, thereby activating SNF1-related kinases (*SnRK2s*) to phosphorylate and activate ABFs [65]. A study on the heat-tolerant rice cultivar “Annapurna” showed that *PP2Cs* and *ABFs* were upregulated at 37 °C temperature stress [22]. In agreement, all the *PP2Cs* and most *ABFs* were upregulated by heat stress in *C. tangshen*, indicating the critical role of the ABA signaling pathway in *C. tangshen* thermotolerance. These results suggest that different hormone signaling pathways are involved in the thermotolerance of *C. tangshen*. Several functional studies indicate that ABF genes could play a remarkable role in improving plants’ stress tolerance. For instance, overexpression of *OsABF1* positively regulated drought tolerance in rice [66]. Additionally, overexpression of *IbABF4* from sweet potatoes could improve heat tolerance in *Arabidopsis* [67]. Thus, the ABF genes identified in this study could play a crucial role in improving the heat tolerance of *C. tangshen.*

### 4.5. DEGs Related to Transcription Factors

Transcriptional factors, including MYBs, NACs, WRKY, bHLH, and bZIP, play a pivotal role in heat stress response [68,69,70,71,72]. In this study, 17 MYBs, 14 NACs, 9 WRKY, 3 bHLH, and 10 bZIP were significantly regulated in response to heat stress in *C. tangshen*. Overexpression of crucial stress-responsive TFs could remarkably improve stress tolerance in plants. For instance, *MYB305*, isolated from *Lilium longiflorum*, with transactivation ability in yeast and plant cells, was reported to play a positive role in thermotolerance [73]. In *Arabidopsis*, *AtWRKY25*, *AtWRKY26*, and *AtWRKY33* were involved in resistance against pathogenic bacteria and abiotic stresses, including heat stress [74]. Likewise, *NAC19* and *bZIP28* identified from *Arabidopsis* positively regulated thermotolerance [69,72]. Overexpression of *AtMYB68* enhanced drought and heat tolerance in *Arabidopsis* and *Brassica napus* [75]. Additionally, overexpression of *OsbZIP46* could positively regulate drought tolerance in rice [76]. In a recent study, a maize NAC TF, *ZmNAC074*, improved heat tolerance in *Arabidopsis* [77]. Thus, we speculated that the TFs identified in this study could play a significant role in improving the thermotolerance of *C. tangshen*.

Additionally, a large number of calcium-dependent protein kinases (CDPKs) were upregulated in response to heat stress in *C. tangshen*. CDPK proteins play crucial roles in plants’ abiotic stress response by regulating downstream target proteins [78]. For instance, overexpression of *CDPK6* enhanced salt and drought tolerance in transgenic *Arabidopsis* [79]. In rice, *OsCDPK7* positively regulates cold, drought, and salinity stresses [80]. Additionally, *ZmCDPK7* was involved in improving heat tolerance in maize [81]. These results indicate that CDPK proteins are a central player in multiple stress responses. Thus, the upregulation of CDPKs could be one of the mechanisms of thermotolerance in *C. tangshen.* In addition, candidate CDPKs identified in this study can be used for functional analysis in response to heat stress.

## 5. Conclusions

This study demonstrated how *C. tangshen* responded to short-term heat stress at the transcriptome level. Heat stress is a significant factor limiting the growth and development of *C. tangshen*. Heat stress reprogrammed the transcriptomic profiles of *C. tangshen* and regulated various genes related to the HSP-HSF network, ROS-scavenging activity, hormone signaling transduction, and TFs. Overall, these findings could provide essential transcriptional information for improving the thermotolerance of *C. tangshen*. Further functional studies are needed to understand the detailed molecular mechanisms of these candidate genes in response to heat stress in *C. tangshen*.

## Figures and Tables

**Table 1:** Statistical analysis of the sequencing data.

Sample	Total Reads	Total Bases	Clean Reads	Clean Bases	Q20 Rate (%)	Q30 Rate (%)	GC (%)
CK-1	45,863,972	6,879,595,800	44,915,008	6,680,680,398	97.85	93.46	44.94
CK-2	47,993,664	7,199,049,600	47,205,020	7,025,130,948	98.10	94.05	44.77
CK-3	53,683,124	8,052,468,600	52,814,730	7,734,007,771	98.03	93.87	44.67
average	49,180,253	7,377,038,000	48,311,586	7,146,606,372	97.99	93.79	44.79
Ht-1	51,810,464	7,771,569,600	50,974,272	7,534,318,727	98.02	93.85	45.00
Ht-2	45,663,096	6,849,464,400	44,953,364	6,625,104,742	98.10	94.07	45.65
Ht-3	50,863,920	7,629,588,000	49,951,766	7,400,820,966	98.22	94.44	45.24
average	49,445,827	7,416,874,000	48,626,467	7,186,748,145	98.11	94.12	45.30

**Table 2:** Statistics of gene annotation by databases.

Database	KOG	KEGG	NR	Swiss-Prot	GO	Total Unigenes	Overall Annotated
Gene Number	19,218	12,563	30,131	26,193	19,546	32,719	30,464
Annotation Ratio	0.5874	0.3840	0.9209	0.8005	0.5974	-	0.9311

**Table 3:** The upregulated CDPKs genes in heat treatment compared with CK.

Gene ID	Log2 Ratio	*p*-Value	q Value	Gene Description
PB.23444.1	3.0345	0.00016	0.001745	Calcium-dependent protein kinase 1 OS = *Oryza sativa* subsp. *japonica* OX = 39947 GN = CPK1 PE = 2 SV = 1
PB.16020.1	10.482	5.72 × 10^−18^	2.04 × 10^−16^	Calcium-dependent protein kinase 8 OS = *Arabidopsis thaliana* OX = 3702 GN = CPK8 PE = 1 SV = 1
PB.1840.1	1.1687	0.007945	0.049257	Calcium-dependent protein kinase 1 OS = *Oryza sativa* subsp. *japonica* OX = 39947 GN = CPK1 PE = 2 SV = 1
PB.15694.1	7.6079	1.11 × 10^−6^	1.79 × 10^−5^	Calcium-dependent protein kinase 8 OS = *Arabidopsis thaliana* OX = 3702 GN = CPK8 PE = 1 SV = 1
PB.13451.1	3.0578	9.96 × 10^−11^	2.45 × 10^−9^	Calcium-dependent protein kinase 1 OS = *Arabidopsis thaliana* OX = 3702 GN = CPK1 PE = 1 SV = 1
PB.12258.1	1.0192	0.00019	0.002038	Calcium-dependent protein kinase 30 OS = *Arabidopsis thaliana* OX = 3702 GN = CPK30 PE = 1 SV = 1
PB.13498.1	1.0004	7.03 × 10^−11^	1.75 × 10^−9^	Calcium-dependent protein kinase 8 OS = *Arabidopsis thaliana* OX = 3702 GN = CPK8 PE = 1 SV = 1
PB.23444.1	3.0345	0.00016	0.001745	Calcium-dependent protein kinase 1 OS = *Oryza sativa* subsp. *japonica* OX = 39947 GN = CPK1 PE = 2 SV = 1
PB.16020.1	10.482	5.72 × 10^−18^	2.04 × 10^−16^	Calcium-dependent protein kinase 8 OS = *Arabidopsis thaliana* OX = 3702 GN = CPK8 PE = 1 SV = 1
PB.32683.1	2.4636	1.97 × 10^−6^	3.06 × 10^−5^	Calcium-binding protein CML24 OS = *Arabidopsis thaliana* OX = 3702 GN = CML24 PE = 2 SV = 2
PB.25136.1	1.7180	2.06 × 10^−8^	4.12 × 10^−7^	Probable calcium-binding protein CML46 OS = *Arabidopsis thaliana* OX = 3702 GN = CML46 PE = 1 SV = 1
PB.1840.1	1.1687	0.007945	0.049257	Calcium-dependent protein kinase OS = *Oryza* *sativa* subsp. *japonica* OX = 39947 GN = CPK1 PE = 2 SV = 1
PB.31428.1	5.0403	3.81 × 10^−70^	4.27 × 10^−68^	Probable calcium-binding protein CML40 OS = *Arabidopsis thaliana* OX = 3702 GN = CML40 PE = 2 SV = 1
PB.31509.1	8.2027	0	0	Probable calcium-binding protein CML44 OS = *Arabidopsis thaliana* OX = 3702 GN = CML44 PE = 2 SV = 2
PB.15694.1	7.6079	1.11 × 10^−6^	1.79 × 10^−5^	Calcium-dependent protein kinase 8 OS = *Arabidopsis thaliana* OX = 3702 GN = CPK8 PE = 1 SV = 1
PB.13451.1	3.0578	9.96 × 10^−11^	2.45 × 10^−9^	Calcium-dependent protein kinase 1 OS = *Arabidopsis thaliana* OX = 3702 GN = CPK1 PE = 1 SV = 1
PB.12258.1	1.0192	0.00019	0.002038	Calcium-dependent protein kinase 30 OS = *Arabidopsis thaliana* OX = 3702 GN = CPK30 PE = 1 SV = 1
PB.14842.1	3.9140	1.78 × 10^−241^	7.04 × 10^−239^	Calcium-dependent protein kinase 8 OS = *Arabidopsis thaliana* OX = 3702 GN = CPK8 PE = 1 SV = 1
PB.13498.1	1.0004	7.03 × 10^−11^	1.75 × 10^−9^	Calcium-dependent protein kinase 8 OS = *Arabidopsis thaliana* OX = 3702 GN = CPK8 PE = 1 SV = 1

**Table 4:** The upregulated bZIP TFs in heat treatment compared with CK.

Gene ID	Log2 Ratio	*p*-Value	q-Value	Gene Description
PB.6276.1	3.4312	1.57 × 10^−80^	2.10 × 10^−78^	bZIP transcription factor 17 OS = *Arabidopsis thaliana* OX = 3702 GN = BZIP17 PE = 1 SV = 2
PB.22986.1	3.3479	1.30 × 10^−8^	2.66 × 10^−7^	ABSCISIC ACID-INSENSITIVE 5-like protein 5 OS = Arabidopsis thaliana OX = 3702 GN = ABF2 PE = 1 SV = 1
PB.21759.1	3.2678	2.75 × 10^−87^	3.94 × 10^−8^	bZIP transcription factor 17 OS = *Arabidopsis thaliana* OX = 3702 GN = BZIP17 PE = 1 SV = 2
PB.19784.1	2.9457	4.47 × 10^−15^	1.40 × 10^−13^	bZIP transcription factor 17 OS = *Arabidopsis thaliana* OX = 3702 GN = BZIP17 PE = 1 SV = 2
PB.19784.1	2.9457	4.47 × 10^−15^	1.40 × 10^−13^	bZIP transcription factor 17 OS = *Arabidopsis thaliana* OX = 3702 GN = BZIP17 PE = 1 SV = 2
PB.19784.1	2.9457	4.47 × 10^−15^	1.40 × 10^−13^	bZIP transcription factor 17 OS = *Arabidopsis thaliana* OX = 3702 GN = BZIP17 PE = 1 SV = 2
PB.30413.1	2.2121	1.54 × 10^−50^	1.27 × 10^−48^	bZIP transcription factor 53 OS = *Arabidopsis thaliana* OX = 3702 GN = BZIP53 PE = 1 SV = 1
PB.20861.1	1.1184	6.48 × 10^−8^	1.22 × 10^−6^	Probable transcription factor PosF21 OS = *Arabidopsis thaliana* OX = 3702 GN = POSF21 PE = 2 SV = 1

**Table 5:** The upregulated NAC TFs in heat treatment compared with CK.

Gene ID	Log2 Ratio	*p*-Value	q-Value	Gene Description
PB.15241.1	8.3623	1.41 × 10^−9^	3.15 × 10^−8^	NAC domain-containing protein 91 OS = *Arabidopsis thaliana* OX = 3702 GN = NAC091 PE = 1 SV = 1
PB.12957.1	8.1420	6.04 × 10^−10^	1.40 × 10^−8^	NAC domain-containing protein 78 OS = *Arabidopsis thaliana* OX = 3702 GN = NAC078 PE = 2 SV = 2
PB.31443.1	4.7942	1.32 × 10^−91^	2.01 × 10^−89^	NAC domain-containing protein 2 OS = *Arabidopsis thaliana* OX = 3702 GN = NAC002 PE = 1 SV = 2
PB.25504.1	4.3026	1.63 × 10^−230^	5.91 × 10^−228^	NAC domain-containing protein 2 OS = *Solanum lycopersicum* OX = 4081 GN = NAP2 PE = 2 SV = 1
PB.27680.1	3.6323	2.95 × 10^−73^	3.49 × 10^−71^	NAC domain-containing protein JA2L OS = *Solanum lycopersicum* OX = 4081 GN = JA2L PE = 2 SV = 1
PB.29002.1	3.3875	7.42 × 10^−156^	1.85 × 10^−153^	NAC domain-containing protein 2 OS = *Arabidopsis thaliana* OX = 3702 GN = NAC002 PE = 1 SV = 2
PB.29160.1	3.1996	9.66 × 10^−225^	3.41 × 10^−222^	NAC domain-containing protein 2 OS = *Arabidopsis thaliana* OX = 3702 GN = NAC002 PE = 1 SV = 2
PB.12308.1	2.6077	2.87 × 10^−82^	3.91 × 10^−80^	NAC domain-containing protein 78 OS = *Arabidopsis thaliana* OX = 3702 GN = NAC078 PE = 2 SV = 2
PB.17150.1	1.7393	0.007178	0.045244	NAC domain-containing protein 17 OS = *Arabidopsis thaliana* OX = 3702 GN = NAC017 PE = 2 SV = 1
PB.12444.1	1.4019	3.30 × 10^−14^	9.93 × 10^−13^	NAC domain-containing protein 82 OS = *Arabidopsis thaliana* OX = 3702 GN = NAC082 PE = 1 SV = 1
PB.23584.1	1.2042	5.20 × 10^−13^	1.48 × 10^−11^	NAC domain-containing protein 53 OS = *Arabidopsis thaliana* OX = 3702 GN = NAC053 PE = 2 SV = 1

**Table 6:** The upregulated WRKY TFs in heat treatment compared with CK.

Gene ID	Log2 Ratio	*p*-Value	q-Value	Gene Description
PB.28576.1	2.1307	4.14 × 10^−5^	0.000509	Probable WRKY transcription factor 41 OS = *Arabidopsis thaliana* OX = 3702 GN = WRKY41 PE = 2 SV = 2
PB.9306.1	2.7500	7.58 × 10^−15^	2.35 × 10^−13^	Probable WRKY transcription factor 46 OS = *Arabidopsis thaliana* OX = 3702 GN = WRKY46 PE = 1 SV = 1
PB.26632.1	1.2038	8.50 × 10^−12^	2.24 × 10^−10^	Probable WRKY transcription factor 40 OS = *Arabidopsis thaliana* OX = 3702 GN = WRKY40 PE = 1 SV = 1
PB.17400.1	1.8484	1.52 × 10^−14^	4.65 × 10^−13^	Probable WRKY transcription factor 53 OS = *Arabidopsis thaliana* OX = 3702 GN = WRKY53 PE = 1 SV = 1
PB.27665.1	1.1763	0.000231	0.002425	Probable WRKY transcription factor 40 OS = *Arabidopsis thaliana* OX = 3702 GN = WRKY40 PE = 1 SV = 1
PB.31800.1	1.4388	0.000696	0.006323	Probable WRKY transcription factor 75 OS = *Arabidopsis thaliana* OX = 3702 GN = WRKY75 PE = 2 SV = 1
PB.9501.1	1.2791	2.97 × 10^−5^	0.000375	F-box/LRR-repeat protein 3 OS = *Arabidopsis thaliana* OX = 3702 GN = FBL3 PE = 2 SV = 1
PB.30350.1	2.1258	1.62 × 10^−51^	1.36 × 10^−49^	Probable WRKY transcription factor 70 OS = *Arabidopsis thaliana* OX = 3702 GN = WRKY70 PE = 1 SV = 1
PB.28576.1	2.1307	4.14 × 10^−5^	0.000509	Probable WRKY transcription factor 41 OS = *Arabidopsis thaliana* OX = 3702 GN = WRKY41 PE = 2 SV = 2

**Table 7:** The downregulated bHLH TFs in Heat treatment compared with CK.

Gene ID	Log2 Ratio	*p*-Value	q-Value	Gene Description
PB.28723.1	−1.3956	2.71 × 10^−7^	4.75 × 10^−6^	Transcription factor bHLH30 OS = *Arabidopsis thaliana* OX = 3702 GN = BHLH30 PE = 1 SV = 1
PB.8767.1	−1.6598	3.01 × 10^−17^	1.04 × 10^−15^	Transcription factor bHLH74 OS = *Arabidopsis thaliana* OX = 3702 GN = BHLH74 PE = 1 SV = 1
PB.30938.1	−1.4028	1.41 × 10^−11^	3.69 × 10^−10^	Transcription factor bHLH147 OS = *Arabidopsis thaliana* OX = 3702 GN = BHLH147 PE = 1 SV = 1

## Data Availability

The raw sequencing data have been submitted to the Genome Sequence Archive (GSA) database at the BIG Sub (https://ngdc.cncb.ac.cn/gsub/, accessed on 1 October 2022). The accession number is CRA004261.

## Supplementary Materials

The following supporting information can be downloaded at: https://www.mdpi.com/article/10.3390/life13010168/s1, Figure S1: L_vs_L_Ht_all.go.enrichment.stat; Figure S2: L_vs_L_Ht.pathway_DEG; Figure S3: L_vs_L_Ht_up.kegg.enrichment.stat; Figure S4: L_vs_L_Ht_down.kegg.enrichment.stat; Table S1: Real time PCR primers used in this study; Table S2: The list of all unigenes; Table S3: GO classification of the unigenes; Table S4: L_vs_L_Ht.DEG_up.Annotation; Table S5: L_vs_L_Ht.DEG_down; Table S6: KEGG_classification; Table S7: L_vs_L_Ht_all.go.significant.enrichment; Table S8: L_vs_L_Ht_up.kegg.significant.enrichment; Table S9: L_vs_L_Ht_down.kegg.significant.enrichment.

## References

[1] Xu X.Y., Xu H.Y., Shang Y., Zhu R., Hong X.X., Song Z.H., Yang Z.P. (2021). Development of the general chapters of the Chinese Pharmacopoeia 2020 edition: A review. J. Pharm. Anal..

[2] Wu Q.N., Luo M., Yao X.D., Yu L. (2020). Purification, structural characterization, and antioxidant activity of the COP-W1 polysaccharide from *Codonopsis tangshen* Oliv. Carbohyd. Polym..

[3] Zhang Y.L., Zhou N., Tan J.P. (2017). Preliminary analysis of the quality difference of *Codonopsis pilosula* from different producing areas. Asia-Pac. Tradit. Med..

[4] Zou Y.F., Zhang Y.Y., Paulsen B.S., Rise F., Chen Z.L., Jia R.Y., Li L.X., Song X., Feng B., Tang H.Q. (2020). Structural features of pectic polysaccharides from stems of two species of *Radix Codonopsis* and their antioxidant activities. Int. J. Biol. Macromol..

[5] Liu C., Chen J., Li E.T., Fan Q., Wang D.Y., Li P., Li X.P., Chen X.Y., Qiu S.L., Gao Z.Z. (2015). The comparison of antioxidative and hepatoprotective activities of *Codonopsis pilosula* polysaccharide (CP) and sulfated CP. Int. Immunopharmacol..

[6] Meng Y., Xu Y.J., Chang C., Qiu Z.P., Hu J.J., Wu Y., Zhang B.H., Zheng G.H. (2020). Extraction, characterization and anti-inflammatory activities of an inulin-type fructan from *Codonopsis pilosula*. Int. J. Biol. Macromol..

[7] Yang D.D., Chen Y., Guo F.X., Huang B.T., Okyere S.A., Wang H. (2019). Comparative analysis of chemical composition, antioxidant and antimicrobial activities of leaves, leaf tea and root from *Codonopsis pilosula*. Ind. Crop. Prod..

[8] Liu Y.Q., Zou X.M., Sun G.R., Bao Y.H. (2017). *Codonopsis lanceolata* polysaccharide CLPS inhibits melanoma metastasis via regulating integrin signaling. Int. J. Biol. Macromol..

[9] Gao Z.Z., Zhang C., Jing L.R., Feng M., Li R., Yang Y. (2020). The structural characterization and immune modulation activitives comparison of *Codonopsis pilosula* polysaccharide (CPPS) and selenizing CPPS (sCPPS) on mouse in vitro and vivo. Int. J. Biol. Macromol..

[10] He Y.S., Zhang M.D., Zhou W.X., Ai L.Q., You J.W., Liu H.H., You J.M., Wang H., Wassie M., Wang M. (2019). Transcriptome analysis reveals novel insights into the continuous cropping induced response in *Codonopsis tangshen*, a medicinal herb. Plant Physiol. Biochem..

[11] Niu Y.T., Zhang J., Wang H.Z., Jin L., Du T., Song P.S., Zhao W.L. (2021). Study on the suitability of *Codonopsis pilosula*. China J. Inf. Tradit. Chin. Med..

[12] Liao C.L., You J.W. (2006). The Cultivation Technology of Medicinal Plants in Enshi, Hubei.

[13] Ashraf M. (2021). Thermotolerance in plants: Potential physio-biochemical and molecular markers for crop improvement. Environ. Exp. Bot..

[14] Prasad P.V.V., Pisipati S.R., Mutava R.N., Tuinstra M.R. (2008). Sensitivity of Grain Sorghum to High Temperature Stress during Reproductive Development. Crop. Sci..

[15] Wang X.M., Hou L.J., Lu Y.Z., Wu B.J., Xue G., Liu M.S., Wang J., Sun Q.X., Elizabeth V., Xu S.B. (2018). Metabolic adaptation of wheat grain contributes to a stable filling rate under heat stress. J. Exp. Bot..

[16] Ma G.J., Zhang M.D., Xu J.L., Zhou W.X., Cao L.W. (2020). Transcriptomic analysis of short-term heat stress response in *Pinellia ternata* provided novel insights into the improved thermotolerance by spermidine and melatonin. Ecotoxicol. Environ. Saf..

[17] Parrotta L., Aloisi I., Faleri C., Romi M., Del Duca S., Cai G. (2020). Chronic heat stress affects the photosynthetic apparatus of Solanum lycopersicum L. cv Micro-Tom. Plant Physiol. Biochem..

[18] Collado-González J., Piñero M.C., Otálora G., López-Marín J., del Amor F.M. (2021). Exogenous spermidine modifies nutritional and bioactive constituents of cauliflower (*Brassica oleracea* var. *botrytis* L.) florets under heat stress. Sci. Hort-Amst..

[19] Hasanuzzaman M., Nahar K., Alam M., Roychowdhury R., Fujita M. (2013). Physiological, Biochemical, and Molecular Mechanisms of Heat Stress Tolerance in Plants. Int. J. Mol. Sci..

[20] Sharma P., Jha A., Dubey R., Pessarakli M. (2012). Reactive Oxygen Species, Oxidative Damage, and Antioxidative Defense Mechanism in Plants under Stressful Conditions. J. Bot..

[21] Baron K.N., Schroeder D.F., Stasolla C. (2012). Transcriptional response of abscisic acid (ABA) metabolism and transport to cold and heat stress applied at the reproductive stage of development in *Arabidopsis thaliana*. Plant. Sci..

[22] Sharma E., Borah P., Kaur A., Bhatnagar A., Mohapatra T., Kapoor S., Khurana J.P. (2021). A comprehensive transcriptome analysis of contrasting rice cultivars highlights the role of auxin and ABA responsive genes in heat stress response. Genomics.

[23] Haider S., Iqbal J., Shaukat M., Naseer S., Mahmood T. (2021). The epigenetic chromatin-based regulation of somatic heat stress memory in plants. Plant Gene.

[24] Ohama N., Sato H., Shinozaki K., Yamaguchi-Shinozaki K. (2017). Transcriptional Regulatory Network of Plant Heat Stress Response. Trends Plant Sci..

[25] Sarkar J., Chakraborty U., Chakraborty B. (2021). High-temperature resilience in Bacillus safensis primed wheat plants: A study of dynamic response associated with modulation of antioxidant machinery, differential expression of HSPs and osmolyte biosynthesis. Environ. Exp. Bot..

[26] Richter K., Haslbeck M., Buchner J. (2010). The Heat Shock Response: Life on the Verge of Death. Mol. Cell.

[27] Dobrá J., Černý M., Štorchová H., Dobrev P., Skalák J., Jedelský P.L., Lukšanová H., Gaudinová A., Pešek B., Malbeck J. (2015). The impact of heat stress targeting on the hormonal and transcriptomic response in *Arabidopsis*. Plant. Sci..

[28] Waqas M., Shahzad R., Khan A.L., Asaf S., Kim Y.H., Kang S.M., Bilal S., Hamayun M., Lee I.J. (2016). Salvaging effect of triacontanol on plant growth, thermotolerance, macro-nutrient content, amino acid concentration and modulation of defense hormonal levels under heat stress. Plant Physiol. Biochem..

[29] Qi Y.X., Liu Y.B., Rong W.H. (2011). RNA-Seq and its applications: A new technology for transcriptomics. Hereditas.

[30] Liu H.B., Shi J.H., Wu M.K., Xu D.L. (2021). The application and future prospect of RNA-Seq technology in Chinese medicinal plants. J. Appl. Res. Med. Aromat..

[31] Grabherr M., Haas B., Yassour M., Levin J., Thompson D., Amit I., Adiconis X., Fan L., Raychowdhury R., Zeng Q.D. (2011). Full-Length transcriptome assembly from RNA-Seq data without a reference genome. Nat. Biotechnol..

[32] Li B., Dewey C. (2011). RSEM: Accurate transcript quantification from RNA-Seq data with or without a reference genome. BMC Bioinform..

[33] Love M., Huber W., Anders S. (2014). Moderated estimation of fold change and dispersion for RNA-Seq data with DESeq2. Genome Biol..

[34] Wu T.Z., Hu E.Q., Xu S.B., Chen M.J., Guo P.F., Dai Z.H., Feng T.Z., Zhou L., Tang W.L., Zhan L. (2021). clusterProfiler 4.0: A universal enrichment tool for interpreting omics data. Innovation.

[35] de Hoon M.J.L., Imoto S., Nolan J., Miyano S. (2004). Open Source Clustering Software. Bioinformatics.

[36] Saldanha A.J. (2004). Java Treeview-extensible visualization of microarray data. Bioinformatics.

[37] Li H.Y., Hu T., Amombo E., Fu J.M. (2017). Transcriptome profilings of two tall fescue (*Festuca arundinacea*) cultivars in response to lead (Pb) stress. BMC Genomics.

[38] Livak K.J., Schmittgen T.D. (2001). Analysis of Relative Gene Expression Data Using Real-Time Quantitative PCR and the 2−ΔΔCT Method. Methods.

[39] Pal S., Sharma R. (2021). Transcription factors and chaperone proteins play a role in launching a faster response to heat stress and aggregation. Comput. Biol. Chem..

[40] Zhao Q., Zhou L.J., Liu J.C., Du X.X., Asad M.A.U., Huang F.D., Pan G., Cheng F.M. (2018). Relationship of ROS accumulation and superoxide dismutase isozymes in developing anther with floret fertility of rice under heat stress. Plant Physiol. Bioch..

[41] Wan B.L., Lin Y.J., Mou T.M. (2007). Expression of rice Ca_2+_-dependent protein kinases (CDPKs) genes under different environmental stresses. Febs. Lett..

[42] Asgary S., Karimi R., Pour P.M., Heydarpour F., Mostafaei S., Farzaei M.H., Moradi S., Aneva I.Y. (2022). Is consumption of pomegranate supplementation effective on oxidative stress biomarkers including MDA, ox-LDL, POX 1, GPX, TAC, and TBRAS? A systematic review and meta-analysis of randomized controlled trials. Curr. Prob. Cardiol..

[43] Wassie M., Zhang W.H., Zhang Q., Ji K., Cao L.W., Chen L. (2020). Exogenous salicylic acid ameliorates heat stress-induced damages and improves growth and photosynthetic efficiency in alfalfa (*Medicago sativa* L.). Ecotox. Environ. Saf..

[44] Larkindale J., Mishkind M., Vierling E. (2005). Plant Responses to High Temperature. Plant Abiotic. Stress.

[45] Sarkar S., Islam A.K.M.A., Barma N.C.D., Ahmed J.U. (2021). Tolerance mechanisms for breeding wheat against heat stress: A review. S. Afr. J. Bot..

[46] Wen F., Wu X.Z., Li T.J., Jia M.L., Liu X.S., Li P., Zhou X.J., Ji X.X., Yue X.M. (2017). Genome-wide survey of heat shock factors and heat shock protein 70s and their regulatory network under abiotic stresses in *Brachypodium distachyon*. PLoS ONE.

[47] Singh S., Chopperla R., Shingote P., Chhapekar S.S., Deshmukh R., Khan S., Padaria J.C., Sharma T.R., Solanke A.U. (2021). Overexpression of EcDREB2A transcription factor from finger millet in tobacco enhances tolerance to heat stress through ROS scavenging. J. Biotechnol..

[48] Wei S.W., Zhang L., Huo G.T., Ge G.J., Luo L.J., Yang Q.C., Yang X., Long P. (2021). Comparative transcriptomics and metabolomics analyses provide insights into thermal resistance in lettuce (*Lactuca sativa* L.). Sci. Hortic..

[49] Pucciariello C., Banti V., Perata P. (2012). ROS signaling as common element in low oxygen and heat stresses. Plant Physiol. Bioch..

[50] Balla K., Bencze S., Janda T., Veisz O. (2009). Analysis of heat stress tolerance in winter wheat. Acta Agron. Hung..

[51] Gad M., Ron M. (2006). Could Heat Shock Transcription Factors Function as Hydrogen Peroxide Sensors in Plants?. Ann. Bot..

[52] Timperio A.M., Egidi M.G., Zolla L. (2008). Proteomics applied on plant abiotic stresses: Role of heat shock proteins (HSP). J. Proteom..

[53] Wang X.R., Yan B., Shi M., Zhou W., Zekria D., Wang H.Z., Kai G.Y. (2016). Overexpression of a Brassica campestris HSP70 in tobacco confers enhanced tolerance to heat stress. Protoplasma.

[54] Zhao D.Q., Xia X., Su J.H., Wei M.R., Wu Y.Q., Tao J. (2019). Overexpression of herbaceous peony HSP70 confers high temperature tolerance. BMC Genom..

[55] Huang Y.Z., Xuan H.D., Yang C.F., Guo N., Wang H.T., Zhao J.M., Xing H. (2019). GmHsp90A2 is involved in soybean heat stress as a positive regulator. Plant Sci..

[56] Dhakal S., Reiter J.W., Laroche A., Schultz E.A. (2021). Leaf vein pattern response to heat and drought requires genes that influence PINFORMED1 localization and is mimicked by ABA treatment. Environ. Exp. Bot..

[57] Hays D.B., Do J.H., Mason R.E., Morgan G., Finlayson S.A. (2007). Heat stress induced ethylene production in developing wheat grains induces kernel abortion and increased maturation in a susceptible cultivar. Plant Sci..

[58] Larkindale J., Huang B. (2004). Thermotolerance and antioxidant systems in Agrostis stolonifera: Involvement of salicylic acid, abscisic acid, calcium, hydrogen peroxide, and ethylene. J. Plant Physiol..

[59] El-Bassiony A.M., Ghoname ElSayed A., el-awadi M., Fawzy Z., Gruda N. (2012). Ameliorative Effects of Brassinosteroids on Growth and Productivity of Snap Beans Grown Under High Temperature. Gesunde Pflanz.

[60] Hu Y.B., Zhao L.F., Chong K., Wang T. (2008). Overexpression of OsERF1, a novel rice ERF gene, up-regulates ethylene-responsive genes expression besides affects growth and development in *Arabidopsis*. J. Plant Physiol..

[61] He Y.H., Xue J., Li H., Han S.K., Jiao J.Q., Rao J.P. (2020). Ethylene response factors regulate ethylene biosynthesis and cell wall modification in persimmon (*Diospyros kaki* L.) fruit during ripening. Postharvest Biol. Tec..

[62] Zhang H.N., Pan X.L., Liu S.H., Lin W.Q., Li Y.H., Zhang X.M. (2021). Genome-wide analysis of AP2/ERF transcription factors in pineapple reveals functional divergence during flowering induction mediated by ethylene and floral organ development. Genomics.

[63] Klay I., Gouia S., Liu M., Mila I., Khoudi H., Bernadac A., Bouzayen M., Pirrello J. (2018). Ethylene Response Factors (ERF) are differentially regulated by different abiotic stress types in tomato plants. Plant Sci..

[64] Choi H.I., Hong J.H., Ha J.O., Kang J.Y., Kim S.Y. (2000). ABFs, a Family of ABA-responsive Element Binding Factors*. J. Biol. Chem..

[65] Zhu J.K. (2016). Abiotic Stress Signaling and Responses in Plants. Cell.

[66] Zhang C.Y., Li C., Liu W.J., Lv Y.D., Yu C.S., Li H.Y., Zhao T., Liu B. (2017). The OsABF1 transcription factor improves drought tolerance by activating the transcription of COR413-TM1 in rice. J. Exp. Bot..

[67] Wang W.B., Qiu X.P., Yang Y.X., Kim H.S., Jia X.Y., Yu H., Kwak S.S. (2019). Sweetpotato bZIP Transcription Factor IbABF4 Confers Tolerance to Multiple Abiotic Stresses. Front. Plant Sci..

[68] Li J.B., Zhao S., Yu X., Du W., Li H., Sun Y., Sun H., Ruan C.J. (2021). Role of Xanthoceras sorbifolium MYB44 in tolerance to combined drought and heat stress via modulation of stomatal closure and ROS homeostasis. Plant Physiol. Bioch..

[69] Guan Q.M., Yue X.L., Zeng H.T., Zhu J.H. (2014). The Protein Phosphatase RCF2 and Its Interacting Partner NAC019 Are Critical for Heat Stress-Responsive Gene Regulation and Thermotolerance in *Arabidopsis*. Plant Cell.

[70] Chen C.H., Chen X.Q., Han J., Lu W.L., Ren Z.H. (2020). Genome-wide analysis of the WRKY gene family in the cucumber genome and transcriptome-wide identification of WRKY transcription factors that respond to biotic and abiotic stresses. BMC Plant Biol..

[71] Koini M.A., Alvey L., Allen T., Tilley C.A., Harberd N.P., Whitelam G.C., Franklin K.A. (2009). High Temperature-Mediated Adaptations in Plant Architecture Require the bHLH Transcription Factor PIF4. Curr. Biol..

[72] Srivastava R., Deng Y., Howell S. (2014). Stress sensing in plants by an ER stress sensor/transducer, bZIP28. Front. Plant Sci..

[73] Wu Z., Li T., Liu X.Y., Yuan G.Z., Hou H.Z., Teng N.J. (2021). A novel R2R3-MYB transcription factor LlMYB305 from *Lilium longiflorum* plays a positive role in thermotolerance via activating heat-protective genes. Environ. Exp. Bot..

[74] Fu Q.T., Yu D. (2010). Q Expression profiles of *AtWRKY25*, *AtWRKY26* and *AtWRKY33* under abiotic stresses. Yi Chuan.

[75] Deng M.D., Wang Y., Kuzma M., Chalifoux M., Tremblay L., Yang S.J., Ying J.F., Sample A., Wang H.M., Griffiths R. (2020). Activation tagging identifies *Arabidopsis* transcription factor AtMYB68 for heat and drought tolerance at yield determining reproductive stages. Plant J..

[76] Tang N., Zhang H., Li X.H., Xiao J.H., Xiong L.Z. (2012). Constitutive activation of transcription factor OsbZIP46 improves drought tolerance in rice. Plant Physiol..

[77] Xi Y., Ling Q.Q., Zhou Y., Liu X., Qian Y.X. (2022). ZmNAC074, a maize stress-responsive NAC transcription factor, confers heat stress tolerance in transgenic *Arabidopsis*. Front. Plant Sci..

[78] Schulz P., Herde M., Romeis T. (2013). Calcium-dependent protein kinases: Hubs in plant stress signaling and development. Plant Physiol..

[79] Xu J., Tian Y.S., Peng R.H., Xiong A.S., Zhu B., Jin X.F., Gao F., Fu X.Y., Hou X.L., Yao Q.H. (2010). *AtCPK6*, a functionally redundant and positive regulator involved in salt/drought stress tolerance in *Arabidopsis*. Planta.

[80] Saijo Y., Hata S., Kyozuka J., Shimamoto K., Izui K. (2000). Over-expression of a single Ca_2+_-dependent protein kinase confers both cold and salt/drought tolerance on rice plants. Plant J..

[81] Zhao Y.L., Du H.W., Wang Y.K., Wang H.L., Yang S.Y., Li C.H., Chen N., Yang H., Zhang Y.H., Zhu Y. (2021). The calcium-dependent protein kinase ZmCDPK7 functions in heat-stress tolerance in maize. J. Integr. Plant Biol..

