# Supplementary material for: Transcriptomic Analysis Provides Novel Insights into the Heat Stress-Induced Response in Codonopsis tangshen
Source: Life (Basel). 2023 Jan 6;13(1):168. doi: 10.3390/life13010168 (PMC9867074; doi:10.3390/life13010168)
Supplement: Supplementary file 1 [file life-13-00168-s001.zip › Table S1 Real time PCR primers used in this study.pdf]

Table S1 Real time PCR primers used in this study

Gene	Forward sequence (5'-3')	Reverse sequence (5'-3')
HSP	CGACTGGAAGGAAACACCGT	CACTTGTCGCCCTCTACCTC
HSF	TTGCCTGATGATGGCGAAGT	AGCTTGACGTCCTTGCAACT
GST	GCAAGACGATGGGGTTTGTC	TCAAGTTGGGGCACTCTGTC
ERF	GCTCTGGTTCGACTGTGACT	TTGCCAGCATCGTCAACTCT
ABF	TGTCACCTGCACCTTACGTC	CAGCCGACTCCCTGTTCTTT
LHC	TTGCTGACCACCTTGCTGAC	AGAGTTTCAGAGAGCCAACCG
Bhlh	TCGATGCACTCCACCAAGTC	GTTCGTTAGAATCGCACGGC
Bzip	TGACAGCAGAAAACCGTCCA	TTGTCGACCGATGTTCCAGG
MYB	ACTACTCAACGGTCGCACTG	CGGACTATCAGGACTCACGC
NAV	CGGTTGGGATCAAGAAGGCT	GTTCTTCTTTCGAGCCGAGC
GAPDH	GGACCGAATGGCTGTTCGTA	CACTGCACCAGGACGAAGAT
