# Supplementary figures and images for: Transcriptomic Analysis Provides Novel Insights into the Heat Stress-Induced Response in Codonopsis tangshen
Source: Life (Basel). 2023 Jan 6;13(1):168. doi: 10.3390/life13010168 (PMC9867074; doi:10.3390/life13010168)

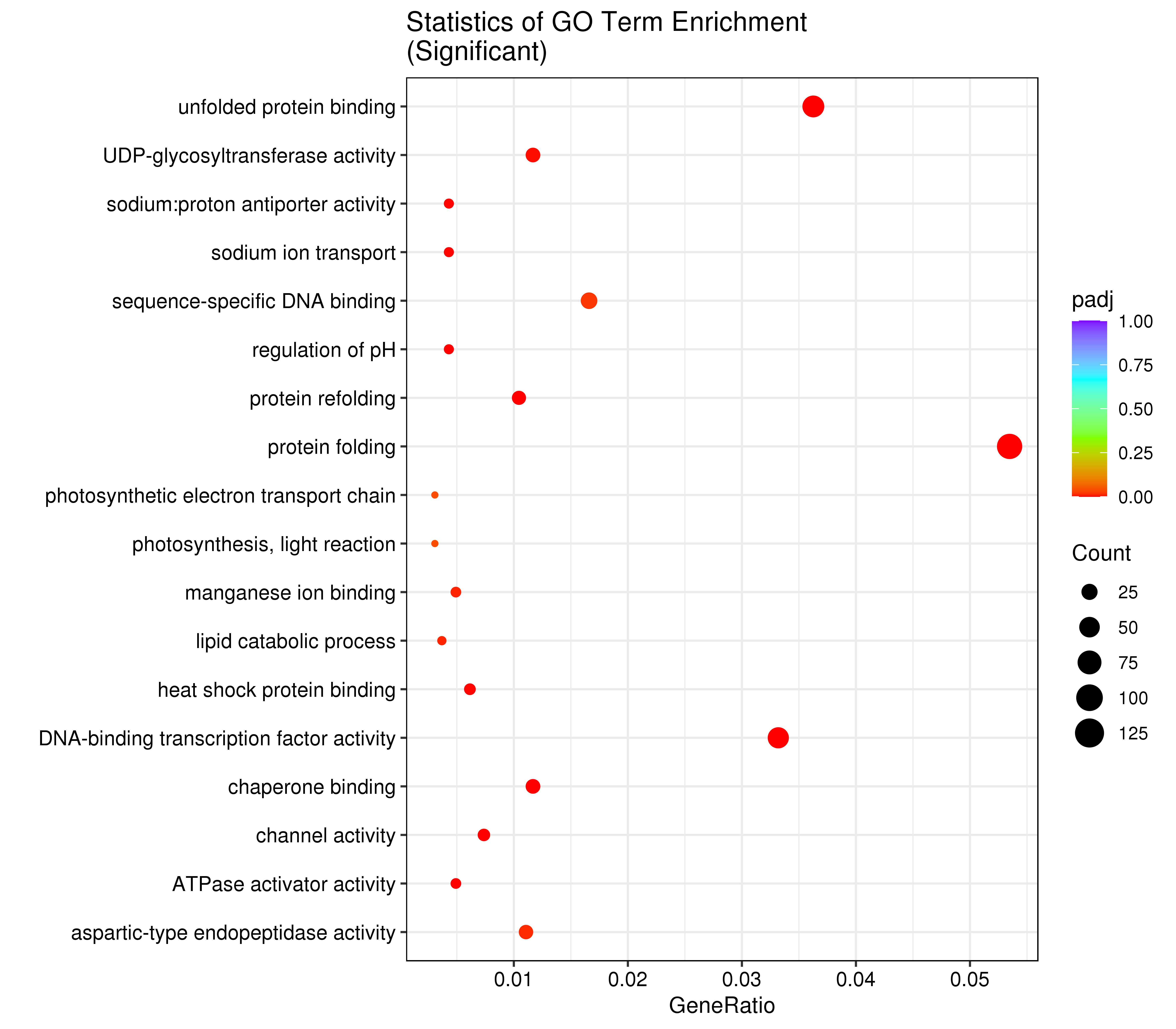

Supplement: Supplementary file 1 [file life-13-00168-s001.zip › Figure S1 L_vs_L_Ht_all.go.enrichment.stat.png]

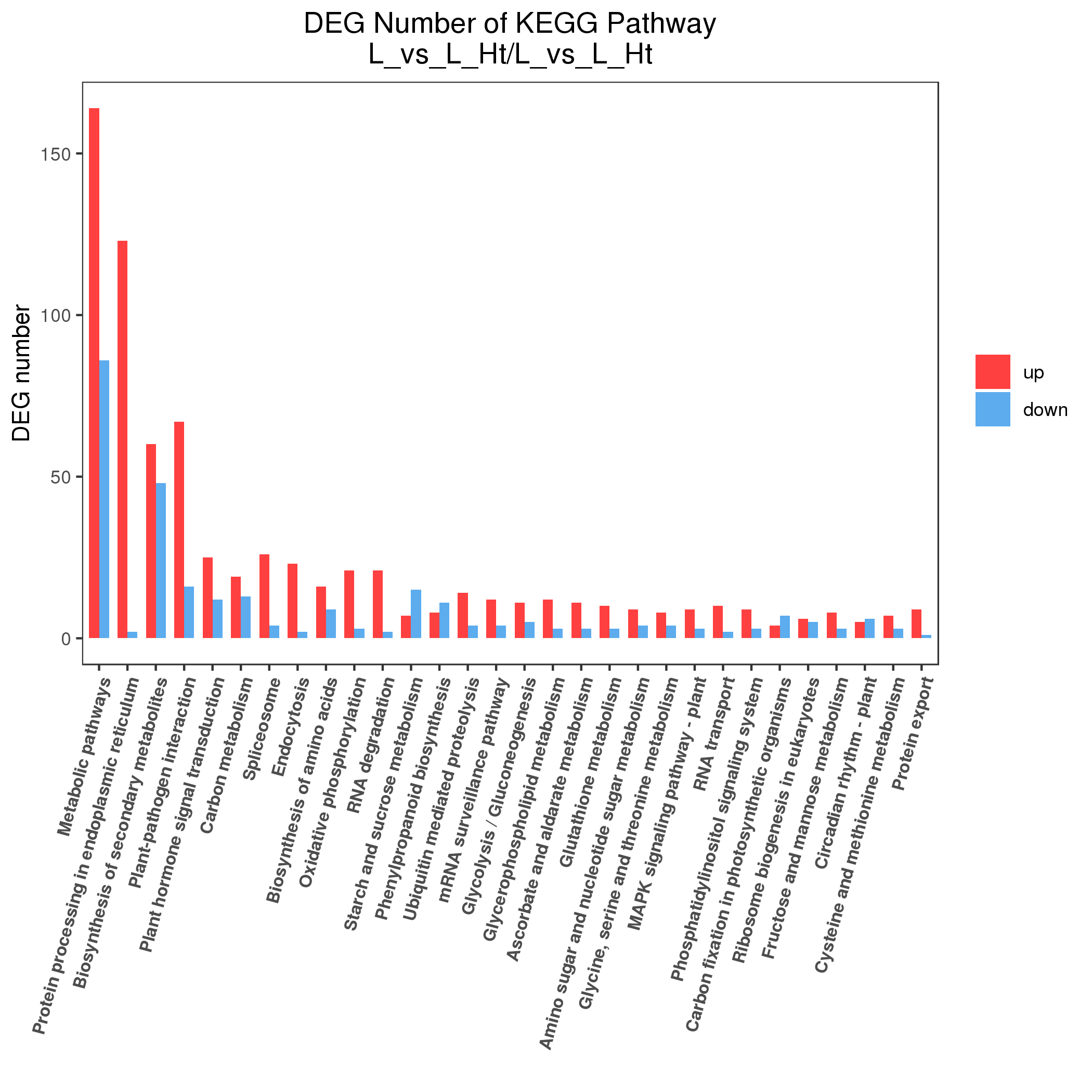

Supplement: Supplementary file 1 [file life-13-00168-s001.zip › Figure S2 L_vs_L_Ht.pathway_DEG.png]

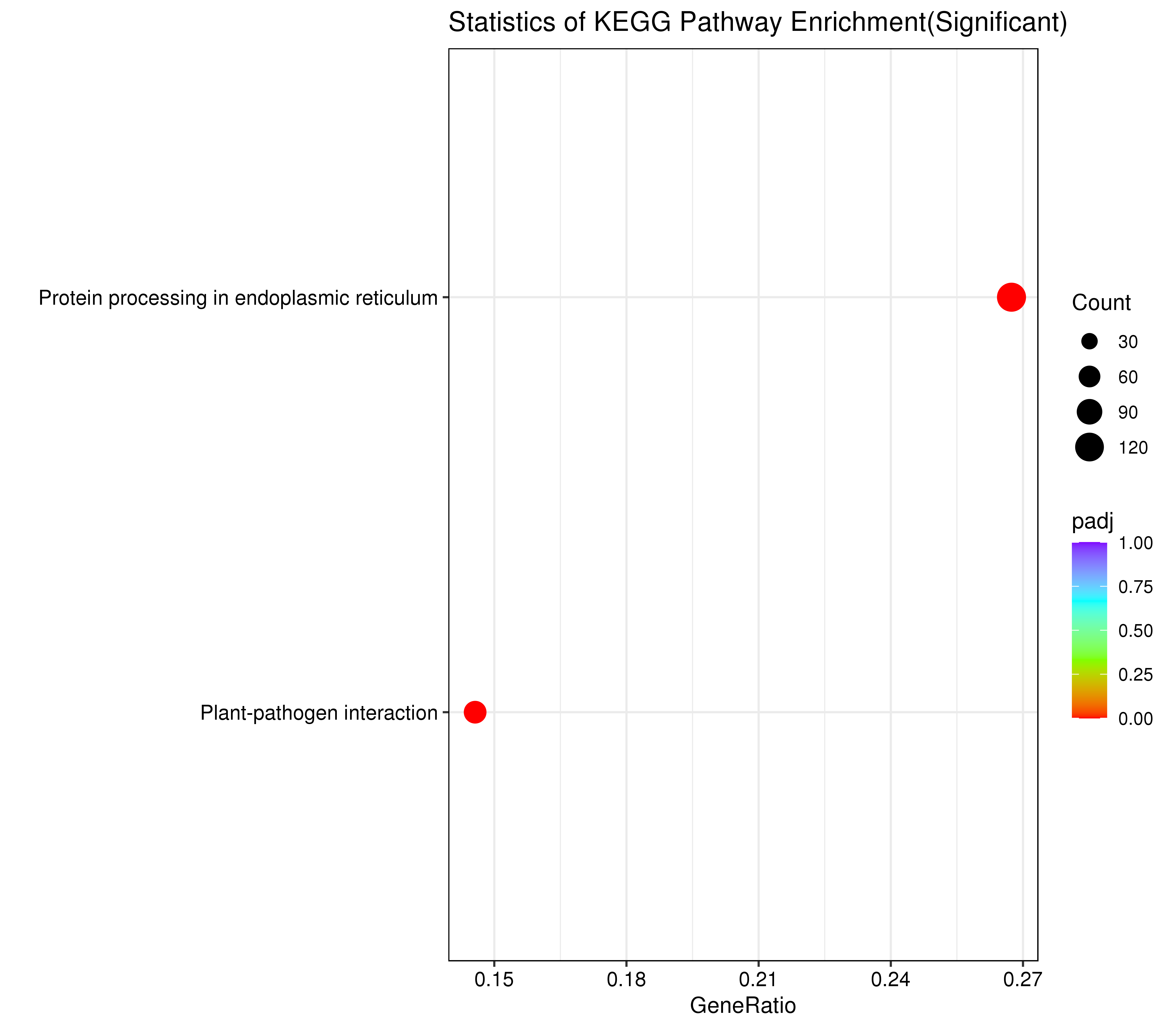

Supplement: Supplementary file 1 [file life-13-00168-s001.zip › Figure S3 L_vs_L_Ht_up.kegg.enrichment.stat.png]

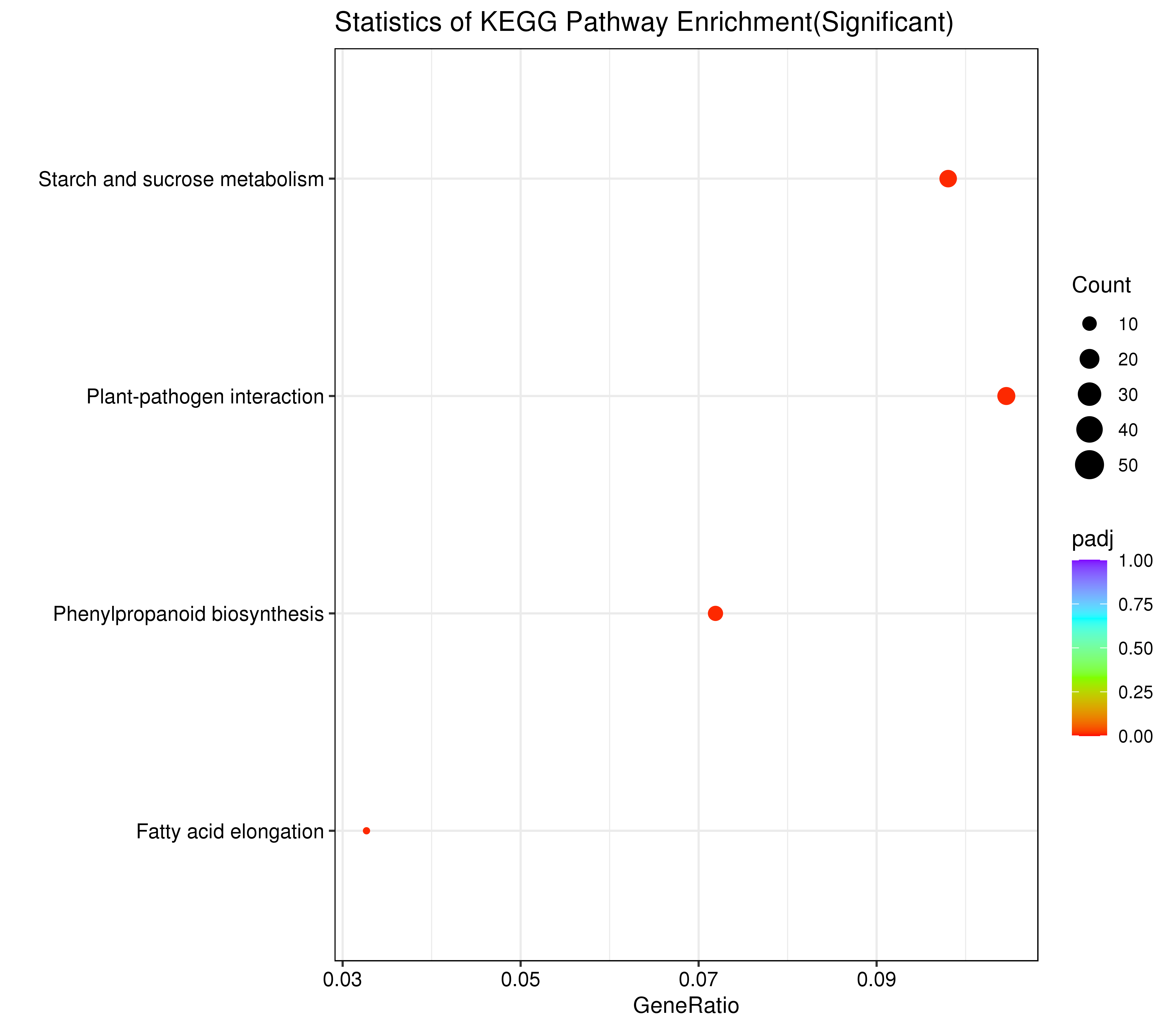

Supplement: Supplementary file 1 [file life-13-00168-s001.zip › Figure S4 L_vs_L_Ht_down.kegg.enrichment.stat.png]
